# Proceedings of the Suffolk District Medical Society

**Published:** 1878-11

**Authors:** 


### PROCEEDINGS OF THE SUFFOLK DISTRICT MEDICAL SOCIETY.

March 30, 1878.—Seventy-five members were present, the president, Dr. Homans, in the chair. The records of the last meeting were read and accepted.

*United States Pharmacopœia.*—It was voted that the councilors be requested to appoint a committee to report upon the next revision of the Pharmacopœia.

*Carcinoma of the Conjunctiva.*—Dr. Albert N. Blodgett read a paper on "Carcinoma of the Coujunctiva," in a case occurring in the practice of Dr. H. Derby, who supplied the clinical history as follows: The patient is a clergyman, seventy-one years of age, native of Ireland. His mother is said to have died of cancer, but no definite clinical history of her case is known. He himself has been in feeble health for a number of years, has had his feet frozen, and is the subject of hernia. September 24,

1877, he presented himself, and complained of a small tumor on the conjunctiva of the left eye, situated at the inner edge and opposite the middle of left cornea, 2 mm. long, 1.5 mm. broad, and 1.5 mm. in height, of a peculiar whiteness, and having a granular surface, as if made up of numerous fine grains of sago. A livid redness surrounded it, gradually shading off into the healthy conjunctival white. In a fortnight later the growth was 4 mm. long, 3 mm. broad and 2.5 mm. high. Ether was administered, and the whole carefully excised October 8th. Healing was good. Two weeks later a very small similar growth was observed at the lower edge of the wound, and after consultation with Dr. Wadsworth, was carefully excised under ether. Every trace of the growth was removed. The cornea remained clear and intact.

Dr. Blodgett gave a description of the histological character of the growth, which was carcinomatous, the microscopic view being represented by India ink drawings. Medical literature contains but few cases where the description of this disease is so clear as to make a diagnosis possible. Many cases are recorded as malignant disease, scirrhus, cancer, epithelial disease, etc., where it is impossible in the present confusion of terms in pathology to determine in many instances what the disease may have been. The importance of a careful histological examination of tumors was pointed out, as well as the necessity of nomenclature in pathological growths which should be founded upon the histological structure of the growth, and not upon physical peculiarities. The proper designation for growths of the kind under consideration is *carcinoma*, rather than any of the names often applied to it, for this name carries with it a distinct idea of the structure of the tumor.

The primary development of carcinoma in a part of the body previously healthy was next described and illustrated by drawings from sections. The epithelium is the invading element in this disease, and forms massive trunks which plow the tissues in all directions, causing their destruction, and producing the enormous loss of substance sometimes observed. The ulceration is caused by the necrosis of the epithelium itself. It is well known that bloood-vessels do not ramify in epithelium, but extend only to the boundary of the connective tissue, upon which the epithelium is developed as a covering. The cells of epithelium are proliferated from its deepest layers, and are pushed up toward the surface by the multiplication of new cells below it. For a certain distance upon its way to the surface the cell can absorb sufficient nourishment from the connective tissue to sustain its vitality, but when it is a little further removed from the connective tissue, the nutritive supply is no longer sufficient, and the cell becomes shrunken, flattened and dry, and is finally cast off from the surface as a scale of epidermis. If the epithelium takes a direction downward into the connective tissue, the march of the epithelial formation is followed by the necrosis of the same at a certain distance behind, thus producing a more and more extensive loss of substance. This may be proved in any advancing case of carcinoma; wherever the disease is active the microscope will show masses of cells penetrating into structures not before infected, and at a certain distance behind we shall find the necrosis of the epithelium as above described. The "bird's nests" are the result of pressure upon the necrosed epithelium, and are not always found. They are not of definite shape, but take any appearance which the pressure of the parts around may impart. The thick, indurated connective tissue around the masses of epithelium is due in great measure to the mechanical irritation of the epithelium, which acts as a foreign body in the tissues. For this reason the Germans call it *Reizungsgewebe* (irritation tissue). In cases of so-called scirrhus it is the hypertrophy and induration of the connectiva tissue surrounding the epithelial masses which cause the great hardness. In secondary deposits the same kind of epithelium is found as in the primary form of the disease.

The probability of recurrence of the disease in carcinoma of the conjunctive seems rather less than in other parts of the body, for the reason that the disease in this location is brought to the notice of the surgeon at a much earlier period of its development than would be thought necessary in other situations. There is now, after a lapse of six months, no sign of a recurrence in this patient, although no one would venture to say there was no danger of a return.

Dr. Wadsworth mentioned two similar cases in which he and Dr. Williams had operated, the eye having been enucleated in one instance, and a tumor, which undoubtedly was an epithelioma, having been removed in the other nine months before without recurrence. He thought that such tumors might not be so rare as had been supposed.

*A New Theory Regarding Myopia.*—Dr. D. Hunt read a paper on myopia, of which a *resume* follows:

Considering the anatomical facts presented at the last meeting, we believe that the form of the myopic eye is caused by increased growth of the brain tissue, which forms the optic vesicle; this increased growth is produced by causes similar to those that have effected the enlargement of the cerebral vesicles, the results of which we notice in the greater frontal development of civilized man.

The type of ciliary muscle characterizing myopic eyes is a production of the same agencies that effect the elongation of the globe; sclerotica and ciliary muscle form from the same layer of embryonic connective tissue, and at the same date.

The separation of the sheaths of the optic nerve represents an embryonic enlargement of the head of the optic nerve, also caused by the increased growth of the retina compsing the optic vesicle.

Posterior staphyloma is the result principally of an increased growth of the embryonic ganglion of the fifth nerve; this ganglion and the optic vesicle enlarge at the expense of the layer of connective tissue situated between them; the posterior portion of the temporal half of the eye is developed in this layer of connective tissue. The augmented growth of the ganglion of the fifth pair, which is the principal agent in the causation of posterior staphyloma, encroaches directly upon the connective tissues forming the root of the first brachial arch. This results in a decrease of the protuberance of the maxillary structures formed from this arch; in other words, contributes to a decrease of prognathism.

Organic lesions that terminate in destruction of nerve terminations are generally not hereditary. We believe that the cause of this peculiarity is to be explained by considering the central nervous system as the active agent in producing hereditary changes; the sudden destruction of the nerve terminations prevents the long-continued and oft-repeated impressions upon the central nervous system that seem to be necessary to make any local peculiarity (the point of origin of the impressions) an hereditary trait.

*Examining Table.*—Dr. Chadwick showed a new table which he had devised for gynæcological purposes. It is described in full, with illustrations, in the *American Journal of Obstetrics*, April, 1878, and is for sale by Messrs. Codman & Shurtleff.

*Cerebral Syphilis.*—Dr. Ayer reported an interesting case of cerebral syphilis, which was published in the *Journal*, September 19, 1878.

Dr. Bowditch asked whether it would not have been allowable to use a mild mercurial course, as the iodide of potassium evidently did not give relief. In this case, when the end was certain death unless relief were obtained, and especially when the case was evidently syphilitic, would not the use of calomel have been proper?

The case reported brought back to Dr. Bowditch's mind that of a student in a seminary of this city, whom he was called to attend. The patient was about twenty years old, and had been gradually growing more and more ill for two years. He had most violent headaches, occurring especially in the night, destroying quiet sleep, and finally reducing not only the physical health, but also the mental powers, so that the patient had been obliged to give up all intellectual exercise, and was listless about everything. When Dr. Bowditch saw him, he was having intense headaches every night, and he seemed childish. He had been under the care of two physicians, one of whom thought he had softening of the brain, and the other diagnosticated a cephalic tumor. On close inquiry, the patient denied all syphilitic taint. There were no symptoms of this disease, unless this nocturnal cephalalgia were one. The patient had been treated during the two years with various remedies without effect. All those employed by Dr. Bowditch seemed useless, and gave no relief. Dr. Bowditch said that our fathers would have used mercurials in such a case, and he thought it proper treatment. Accordingly, discarding all other remedies, calomel alone was given, a grain or two grains daily, in divided doses, three times a day. The patient was directed to suspend temporarily the treatment as soon as any soreness of the mouth, or even a metallic taste, was felt. The result was that, for six weeks or more, the slightest soreness and ptyalism were kept up until the patient wholly recovered. Some relief began with the first trace of the mercury on the mouth, and it went steadily onwards to the perfect cure. It seemed as if something had been effectually overcome by the use of calomel.

Dr. Bowditch was well aware that one case would prove nothing. Dr. E. H. Clarke, when the case was read at another society, had asked whether it might not have been a mere coincidence rather than a consequence of the mercurials. The reply was that it was easier to believe the cure was a consequence of the continued use of the remedy than that, after two years of suffering and gradual prostration in body and mind, with constant headaches, relief, by a mere coincidence, should have suddenly begun, and gone on step bp step, but comparatively quickly, to perfect restoration to health, without any relation existing between the remedy and the cure. Dr. Bowditch regretted that mercurials had not been cautiously tried in the case reported by Dr. Ayer.

